# Overcoming the cytosine base editing barrier: Virus‐like particles with uracil‐DNA glycosylase inhibition unlocking the clinical potential

**DOI:** 10.1002/ctm2.70803

**Published:** 2026-08-27

**Authors:** Yuqing Wu, Xingyu Zhu, Junjie Zhu, Jia Chen, Jiaxu Hong

**Affiliations:** ^1^ Department of Ophthalmology, Eye & ENT Hospital, State Key Laboratory of Brain Function and Disorders, MOE Frontiers Centre for Brain Science Fudan University Shanghai China; ^2^ Gene Editing Centre, School of Life Science and Technology ShanghaiTech University Shanghai China; ^3^ Shanghai Clinical Research and Trial Centre Shanghai China; ^4^ Shanghai Academy of Natural Sciences (SANS) ShanghaiTech University Shanghai China; ^5^ NHC Key Laboratory of Myopia and Related Eye Diseases Shanghai China; ^6^ Shanghai Key Laboratory of Gene Editing and Cell Therapy for Rare Diseases Shanghai Engineering Research Centre of Synthetic Immunology Shanghai China; ^7^ Department of Ophthalmology Jinjiang Second Hospital Jinjiang China; ^8^ Department of Ophthalmology Children's Hospital of Fudan University, National Pediatric Medical Centre of China Shanghai China

1

The vast majority of known genetic pathogenic variants are driven by single base substitutions, particularly A‐to‐G and C‐to‐T transitions (or their respective complementary T‐to‐C and G‐to‐A mutations).^1^ Cytosine base editors (CBEs) and adenine base editors (ABEs) can precisely correct these errors without introducing DNA double‐strand breaks.^2^ However, the clinical translation of in vivo base editing has consistently been constrained by delivery hurdles. Although adeno‐associated viruses (AAVs) are widely used, their prolonged gene expression introduces safety risks, including more severe off‐target editing and the potential for genomic integration.^3^ Lipid nanoparticles (LNPs) offer a transient alternative, yet their efficacy is primarily restricted to the liver.^4^ Virus‐like particles (VLPs) represent a highly promising delivery modality, capable of transiently delivering genome editors as ribonucleoproteins (RNPs).^5^ Moreover, their tissue tropism can be modulated by engineering the envelope glycoproteins, enabling targeted delivery to different tissues, organs, and even specific cell types. While previous studies have successfully established VLP‐mediated in vivo delivery for Cas9 nuclease, ABEs and prime editors, VLP‐mediated CBE delivery had thus far only been demonstrated in vitro.^6^ It remained unclear whether VLPs could achieve meaningful in vivo cytosine base editing.

To address this unmet clinical need, the current study pinpointed the underlying biological barrier: host endogenous UNGs actively excise the edited uracils.^7^ Conventional VLPs deliver a finite, rapidly degrading supply of uracil DNA glycosylase inhibitor (UGI) proteins. Consequently, host DNA repair mechanisms suppress the editing outcomes. By engineering the transformer base editor (tBE) and further optimising the Moloney murine leukaemia virus (MMLV)‐derived VLP system to generate tBE‐VLP4, this strategy successfully enhanced the recruitment and packaging of UGI proteins. This translates into a transient yet robust in vivo editing platform. By overcoming innate repair defences, it ensures stable therapeutic effects.

The rapid onset of action highlights the clinical potential of this system. In a mouse model of tyrosinemia type I, tBE‐VLP4 targeting *mHpd* achieved up to 64.2% editing efficiency in the liver, successfully rescuing the lethal disease phenotype. Notably, treated mice could be safely withdrawn from the protective drug nitisinone (NTBC) just two days post‐VLP injection. For managing clinical metabolic crises, this extremely rapid therapeutic onset demonstrates a distinct advantage over AAV‐mediated therapies, which typically require weeks of bridging therapy due to lagging expression kinetics. Furthermore, tBE‐VLP4 successfully lowered serum cholesterol by efficiently disrupting *mPcsk9* without triggering any metabolic side effects.

Both LNPs and VLPs offer transient expression, reducing off‐target editing and avoiding integration risks. However, while LNPs are largely liver‐restricted, VLPs exhibit broader tissue tropism.^4^ To directly evaluate its tissue versatility, tBE‐VLP4 was further investigated in the eye. Through subretinal injection in a neovascular age‐related macular degeneration model, the system successfully delivered CBE to the retinal pigment epithelium to disrupt *mVegfa*. This intervention significantly reduced pathological choroidal neovascular leakage and partially restored retinal electrophysiological function. Recent reports demonstrate that VLPs incorporating engineered glycoproteins can achieve cell‐type‐specific delivery of Cas9‐nuclease RNPs.^8^ Extending this strategy to tBE‐VLP4 could further expand its therapeutic scope.

Clinical adoption ultimately hinges on safety. In previous studies, dual‐AAV delivery of tBE_V5,^9^ which incorporates an mA3dCDI domain to suppress off‐target activity, yielded no detectable off‐target events. Remarkably, no detectable off‐target activity was observed in vitro or in vivo following VLP delivery of tBE_V1.3, which lacks the mA3dCDI domain. This likely reflects the transient, single‐dose protein delivery achieved by VLPs, where rapid degradation of the editor limits its opportunity to engage off‐target sites compared to AAV delivery.

DNA‐based vehicles like AAV sustain intracellular UGI expression, while LNP‐mRNA platforms enable transient yet robust UGI production.^10^ In contrast, VLPs deliver a finite amount of preformed UGI protein in a single dose. As a result, although VLP delivery enables high‐precision C‐to‐T editing, the effective intracellular dose of the editing machinery may be limited, and rapid UGI protein turnover after single‐dose administration may contribute to modestly lower editing efficiencies compared to AAV or LNP‐mRNA delivery.

Overall, tBE‐VLP4 represents a major conceptual and practical step forward. By concurrently engineering both the base editor and the VLP system to overcome the UNG biological barrier, this work establishes a robust system for efficient and precise in vivo C‐to‐T editing. Beyond specific disease models, its transient nature and superior specificity provide a generalizable framework that can be readily extended to other base editor variants and broader clinical contexts. Furthermore, its compatibility with engineered glycoproteins promises to unlock precise, cell‐type‐specific targeting in the future.

## AUTHOR CONTRIBUTIONS

*Conceptualization*: Jiaxu Hong and Jia Chen. *Writingoriginal draft preparation*: Yuqing Wu. *Writingreview and editing*: Yuqing Wu, Junjie Zhu, Xingyu Zhu, Jiaxu Hong and Jia Chen. *Supervision*: Jiaxu Hong and Jia Chen. *Project administration*: Jiaxu Hong. *Funding acquisition*: Jiaxu Hong. All authors have read and agreed to the published version of the manuscript.

## CONFLICT OF INTEREST STATEMENT

The authors declare no conflict of interest.

## ETHICS STATEMENT

Our study did not involve animal or human clinical trials and was not unethical.

## Data Availability

No new data were created or analyzed in this study. Data sharing is not applicable to this article.
